# PeTMbase: A Database of Plant Endogenous Target Mimics (eTMs)

**DOI:** 10.1371/journal.pone.0167698

**Published:** 2016-12-09

**Authors:** Gökhan Karakülah, Kuaybe Yücebilgili Kurtoğlu, Turgay Unver

**Affiliations:** 1 İzmir International Biomedicine and Genome Institute (iBG-izmir), Dokuz Eylül University, İnciraltı, İzmir, Turkey; 2 Department of Molecular Biology and Genetics, İstanbul Medeniyet University, İstanbul, Turkey; East Carolina University, UNITED STATES

## Abstract

MicroRNAs (miRNA) are small endogenous RNA molecules, which regulate target gene expression at post-transcriptional level. Besides, miRNA activity can be controlled by a newly discovered regulatory mechanism called endogenous target mimicry (eTM). In target mimicry, eTMs bind to the corresponding miRNAs to block the binding of specific transcript leading to increase mRNA expression. Thus, miRNA-eTM-target-mRNA regulation modules involving a wide range of biological processes; an increasing need for a comprehensive eTM database arose. Except miRSponge with limited number of Arabidopsis eTM data no available database and/or repository was developed and released for plant eTMs yet. Here, we present an online plant eTM database, called PeTMbase (http://petmbase.org), with a highly efficient search tool. To establish the repository a number of identified eTMs was obtained utilizing from high-throughput RNA-sequencing data of 11 plant species. Each transcriptome libraries is first mapped to corresponding plant genome, then long non-coding RNA (lncRNA) transcripts are characterized. Furthermore, additional lncRNAs retrieved from GREENC and PNRD were incorporated into the lncRNA catalog. Then, utilizing the lncRNA and miRNA sources a total of 2,728 eTMs were successfully predicted. Our regularly updated database, PeTMbase, provides high quality information regarding miRNA:eTM modules and will aid functional genomics studies particularly, on miRNA regulatory networks.

## Introduction

MicroRNAs (miRNAs) are a class of small RNA (~21 nt) molecules, that has emerged as key regulators of gene expression at post-transcriptional level [1]. In the current molecular framework for miRNA biogenesis, miRNA coding genes (*MIR* genes) produce primary miRNAs (pri-miRNAs) utilizing RNA polymerase II. Pri-miRNAs then undergoes two subsequent cleavage steps by DICER-LIKE1 (DCL1) enzymes to end up with miRNA/miRNA* duplex [2]. Then, the double stranded RNA molecules are loaded onto RNA-induced silencing complex (RISC) which can recognize the specific target mRNA by sequence complementarity in which mature miRNA strand is directed to its target strand and induce silencing mechanism through translational repression or site-specific cleavage of the mRNA molecule [3]. In plants, miRNAs have vital roles in diverse set of biological processes such as growth, development, biotic/abiotic stress responses and signal transduction [4–6].

miRNA activity is also finely regulated by a recently discovered mechanism called endogenous target mimicry (eTM) [7]. Target mimics, also described as miRNA decoys, sponges or competing endogenous RNAs (ceRNAs), are generally belonged to long non-coding RNA (lncRNA) class [8]. LncRNAs are RNA transcripts that are longer than 200 nt in length and lack open reading frame [9]. Despite the small number of lncRNAs examined for their biological functions, findings indicate a regulatory role in gene expression at both transcriptional and post-transcriptional levels [10–12]. Some lncRNAs bind miRNA binding sites and blocks the interaction between miRNA and its specific target mRNA for further regulation. In target mimicry, eTMs bind to miRNAs with a three-nucleotide bulge between the 5' end 10^th^ and 11^th^ positions via sequence complementarity. In this way, the eTM-miRNA pairing rescues the real target transcripts to be cleaved by its complementary miRNA, leading to increased expression levels of target mRNAs. This ceRNA hypothesis that has recently gained attention, presumes the enhancing the expression of corresponding target transcripts through sequestering miRNA activity [13].

The first discovered eTM in plants, *Induced by Phosphate Starvation 1 (IPS1*) transcript was characterized in Arabidopsis as an endogenous lncRNA [14]. With a 3-nt bulge in the miRNA cleavage site, IPS1 binds to phosphate starvation-induced miRNA, ath-miR399. Due to the presence of the loop in base pairing, ath-mir399 cannot bind and specifically cleave the target transcript. Therefore, IPS1 serves as a miRNA target mimic (or decoy) and inhibits binding of ath-miR399 to its target transcript, PHO2. Franco-Zorilla and his colleagues observed that the IPS1 overexpressor plants accumulated increased amounts of target transcript PHO2^14^. Since then, several eTMs for some of the conserved miRNAs have been computationally identified in Arabidopsis, rice and other sequenced plant genomes [15–17]. Artificial eTMs can also induce target mimicry, thus through functional studies, biological roles of miRNAs were revealed by manipulating miRNA activity [18–20].

As this novel gene regulation network plays vital roles in a wide range of biological processes, an increasing need for a comprehensive eTM database arose. Although, a number of lncRNA databases such as TAIR (for Arabidopsis) [21], PlantNATsDB (natural antisense transcripts) [22], lncRNAdb (functional lncRNA database) [10], NONCODE (noncoding RNAs in only Arabidopsis as plant species) [23], PLncDB (with a limited number of lncRNAs in only Arabidopsis) [24], GreeNC (lncRNA repository in plants and algae) [25], CANTATAdb (lncRNA database covering ten plant species) [26] and PNRD for non-coding RNAs in four plant species [27] are available, except miRSponge covering only a very limited number of eTM data for *Arabidopsis thaliana* [28], there is no any comprehensive available database specifically developed for plant eTMs yet. To provide a high quality tool for plant scientist that can accelerate the research on this unique class of transcripts, we established an online database called PeTMbase, which deposits computationally predicted eTMs in plants.

## Materials and Methods for Database Design

### Collection and Processing of Plant Transcriptome Libraries

To build a comprehensive catalogue of lncRNA transcripts from available plant transcriptomes, RNA-sequencing libraries generated from leaf samples of 11 selected monocot/dicot plant species, including *Arabidopsis thaliana*, *Brachypodium distachyon*, *Glycine max* (soybean), *Hordeum vulgare* (barley), *Medicago truncatula*, *Oryza sativa* (rice), *Populus trichocarpa* (poplar), *Sorghum bicolor*, *Solanum lycopersicum* (tomato), *Triticum aestivum* (wheat), and *Zea mays* (maize), were downloaded from Sequence Read Archive (SRA) of National Center for Biotechnology Information (NCBI) [29] by SRA Tool Kit v.2.7.0. Consequently, a total of 21 transcriptome libraries produced by the Illumina sequencing platform were included in the study (S1 Table).

**Fig 1:**
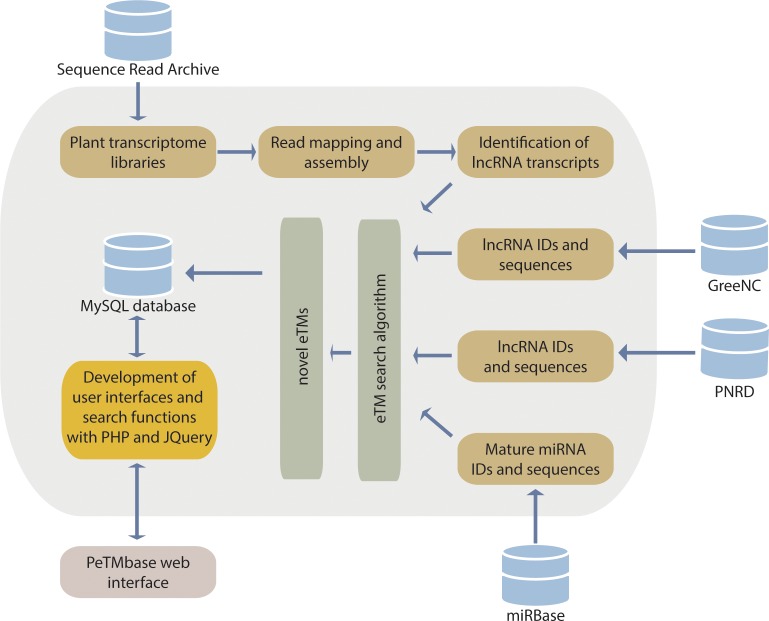
Workflow diagram followed to construct PeTMbase. Raw data sets from diverse public databases were incorporated to extract the biologically relevant information. Sequence libraries were downloaded from SRA database, and known lncRNA sequences retrieved from non-coding RNA databases. MiRNA binding sites within lncRNA sequences were identified with a custom R script, and flexible and user-friendly interfaces were developed diverse scripting languages and web technologies.

The quality graphs of each library were visually examined through SRA browser, and sequence files were converted to FASTQ files using fastq-dump utility of SRA Tool Kit with—skip-technical,—clip and—split-files (for paired end libraries only) options. Sequencing reads in FASTQ files were mapped to the corresponding reference genomes using STAR aligner v2.5.1 [30] with default parameters, and the mean mapping rate of total 21 libraries was found 88.50% (min: 43.64%, max: 94.04%), indicating that the libraries were suitable for performing *ab initio* transcriptome assembly (S2 Table). To build STAR aligner indexes, the corresponding reference genome assemblies in multi-FASTA format were downloaded from the ftp site of Ensembl Plants database release 31 [31]. The *ab inito* transcriptome assembly for each plant species was performed with Cufflinks v2.2.1 [32] to generate a comprehensive catalog of novel lncRNA transcripts. Subsequently, the transcript.gtf files generated in each Cufflinks run were merged with Cuffmerge tool of the Cufflinks suite. To identify novel intergenic transcripts, each merged GTF file was then compared to the corresponding Ensembl v.31 reference annotation using cuffcompare tool. Coding potential of novel transcripts were examined using Transdecoder v.1 (http://transdecoder.github.io/), and sequences longer than 200 nucleotides and containing Open Reading Frame (ORF) < 100 amino acids were classified as novel lncRNAs. We also manually retrieved and included known lncRNA species, annotated by GReeNC [25] and PNRD [27] plant non-coding RNA databases, to generate a complete list of lncRNAs for further analyses. A schematic diagram on the workflow of our study is shown in Fig 1.

### Identification of eTMs

**Fig 2:**
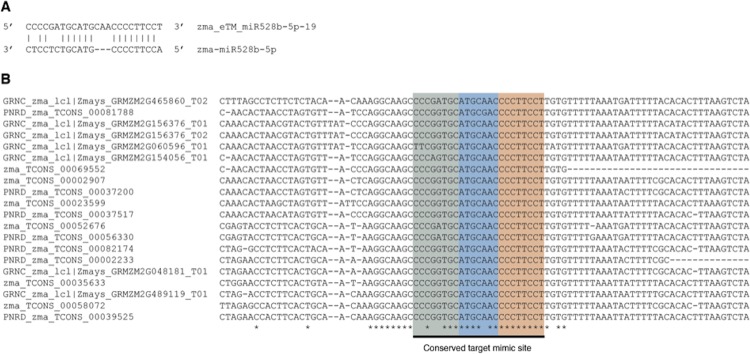
Illustration of an example miRNA:eTM pairing and conserved binding site. Pairing the *Z*. *mays* miRNA, 528b-5p-19. (a) Potential binding sites of plant miRNAs were scanned within novel and known lncRNA sequences using a set of rules previously described by Wu et al[17]. (b) Several conserved miRNA binding sites were identified among eTMs. While the first eight bases (brown-colored) of target mimic site are perfectly preserved across the sequences, there are mismatches within bulge (blue-colored) and tail (green-colored) regions.

A total of 4,323 mature miRNA sequences of 11 plant species were retrieved from miRBase release 21 [33]. To determine putative plant eTMs, possible miRNA target mimic sites were scanned within lncRNA sequences, and marked where they were identified through a custom script written in R programming language [34] as per the following rules previously described in [17]: (i) the 2^nd^ to 8^th^ positions at the 5’ end of a miRNA sequence must perfectly match to target lncRNA sequence, (ii) three unpaired nucleotides (bulges) are allowed between the 9th to 12th positions at the 5’ end of the miRNA sequence, and (iii) at most 3 nucleotide mismatch (excluding bulge region) can be between miRNA and lncRNA sequences. Fig 2A shows an example of pairing *Z*. *mays* eTM, zma_eTM_miR528b-5p-19, and its potential target miRNA, zma-miR528b-5p. Consequently, a large number of lncRNA sequences meeting the binding rules described above were classified as eTMs. Using this approach, several conserved target mimic sites within eTM sequences were discovered among the selected plant species particularly, *A*. *thaliana*, *O*. *sativa*, *S*. *bicolor*, *T*. *aestivum*, and *Z*. *mays* (Fig 2B).

### Database Construction and Development of User Interfaces

To provide a unique and searchable resource of plant eTMs, and to bring currently discovered miRNA mimicry molecules, we established an online database called PeTMbase. Computational analysis of the plant lncRNA sequences from a variety of data sources led to the identification of several novel eTMs in multiple species. Therefore, we have assigned a unique PeTMbase ID for each predicted eTM as follows: (i) the source of eTM sequence is represented by the first three letters of PeTMbase ID. For instance, it is given “zma” prefix if eTM is discovered in ***Z****ea* ***ma****ys*, (ii) the following three letters are “eTM”, and (iii) the rest of PeTMbase ID comprise miRBase ID of target miRNA. In case multiple eTM sequences targeting the same miRNA, incremental integer starting from 1 is suffixed to corresponding PeTMbase ID. The database stores individual information including, PeTMbase ID, sequence, miRNA target(s), transcript properties, and associated external links (if available), for each eTM into the underlying MySQL v5.7 database (https://www.mysql.com/). The user interfaces were developed with Hyper Text Markup Language (HTML) 5.0, and database search operations of PeTMbase were coded in PHP v7.1 (http://www.php.net/). The communication between the user interface of PeTMbase and MySQL database was implemented with PHP scripts. To offer a simple and an easy-to-use searching experience for the users, it was utilized JQuery (https://jquery.com/) AJAX (asynchronous HTTP) methods.

## Database Usage and Utility

### eTM Search by miRNA Name

**Fig 3:**
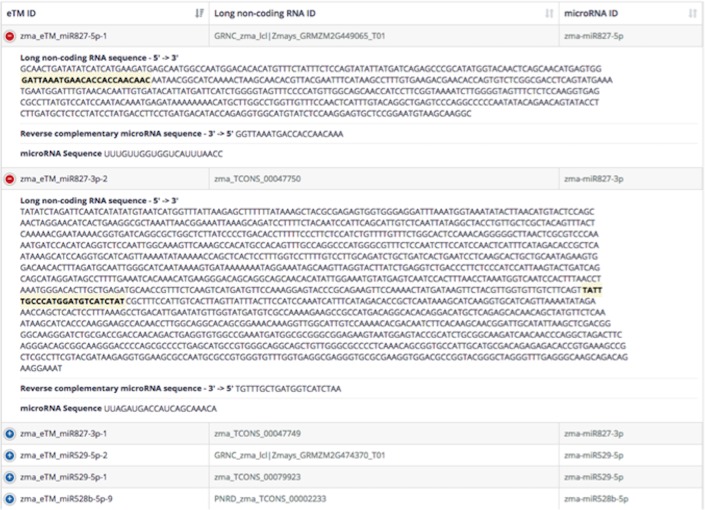
An eTM:lncRNA sequence representation in PeTMbase. The full cDNA sequence of the predicted eTMs, and their target miRNA sequences can be viewed following the database search. miRNA binding site within the eTM sequences is highlighted and the sequence properties of lncRNAs can be retrieved by clicking over the lncRNA ID.

A database of 2,728 eTMs (411 *A*. *thaliana*, 16 *B*. *distachyon*, 487 *G*. *max*, 18 *H*. *vulgare*, 268 *M*. *truncatula*, 431 *O*. *sativa*, 211 *P*. *trichocarpa*, 107 *S*. *bicolor*, 16 *S*. *lycopersicum*, 291 *T*. *aestivum*, and 472 *Z*. *mays*) was developed from both manually retrieved and novel long non-coding RNA transcripts. Using any miRNA name, it is possible to search and retrieve the potential eTM within the lncRNA sequence, which contains miRNA-binding site. Once miRNA name is queried in the database, possible putative eTM entries associated with the miRNA of interest are listed and potential binding site(s) can be visualized by clicking on the PeTMbase IDs. Furthermore, the full cDNA sequence of eTM(s), along with its target miRNA sequence is provided within results window (Fig 3). Additional information regarding transcript properties such as, genomic coordinates and assembly information, and the corresponding miRNA are also supplied. If eTM sequence is retrieved from an external database, PeTMbase directs the user to original data source so that one can view and explore eTM features in-depth.

### eTM Search by Plant Species

PeTMbase also enables a highly flexible user interface to retrieve eTM sequences by choosing the plant species. A certain species can be selected from the drop down menu provided in the interactive interface of the database and all available eTM information is listed in the species selection. Moreover, the user can save detailed search results in comma separated values file (.csv) format, which allows incorporating provided information with other bioinformatics tools such as blast.

## Conclusions and Future Works

Here, we constructed a living database of plant eTMs, which is the most comprehensive eTM repository mining previously sequenced plant transcriptomes. PeTMbase has the following features: i) to search eTM sequences by miRNA name, ii) to search eTM sequences in selected plant species, iii) to retrieve a comprehensive information such as gene ID, exon number, genomic coordinates of the lncRNA and detailed information of the corresponding miRNA sequence, iv) to download eTM data per plant species.

This open-access database enables its users to reach eTM sequences and informs plant researchers on miRNA:eTM modules. Therefore, our up-to-date database will help to conduct functional genomics studies on miRNA and target genes. The modular and extensible architecture of PeTMbase is open to grow and to integrate more plant species as we include more plant species and dissect new RNA-sequencing data. Thus the number of eTMs belonged to a diverse set of plant species is increased. In addition to that, an analysis and a submission module will be incorporated, so that the users will be able to perform eTM prediction by sequence and register their research findings to our public database. PeTMbase will be useful in studies particularly on miRNA regulatory networks and to provide insights into the regulatory roles of eTMs.

## Supporting Information

S1 Table SRA data sets utilized for the identification of novel lncRNA sequences. (PDF)

S2 Table Reference genomes and alignment statistics. Reference genome assemblies utilized for short read mapping and alignment statistics associated with the transcriptome libraries.
(PDF)
