# Supplementary material for: PeTMbase: A Database of Plant Endogenous Target Mimics (eTMs)
Source: PLoS One. 2016 Dec 9;11(12):e0167698. doi: 10.1371/journal.pone.0167698 (PMC5148581; doi:10.1371/journal.pone.0167698)
Supplement: S1 Table — (PDF) [file pone.0167698.s001.pdf]

S1 Table. SRA data sets utilized for the identification of novel lncRNA sequences.

Species	SRA ID	Library type	Library size (bases)	Platform
Arabidopsis thaliana	ERR754084	PE*	3.6G	Illumina HiSeq 2000
Brachypodium distachyon	DRR048605	SE**	2.9G	Illumina HiSeq 2000
Glycine max	SRR3475914	PE	2.7G	Illumina HiSeq 2000
	SRR2079642	SE	9.7G	Illumina HiSeq 2000
	SRR1592302	PE	3.5G	Illumina HiSeq 2500
Hordeum vulgare	ERR674189	PE	3.4G	Illumina HiSeq 1000
Medicago truncatula	SRR1523070	PE	9.6G	Illumina HiSeq 2000
	SRR1523072	PE	9.5G	Illumina HiSeq 2000
	SRR1523077	PE	9.1G	Illumina HiSeq 2000
Oryza sativa	SRR952242	PE	3.2G	Illumina Genome Analyzer II
	ERR687857	PE	3.2G	Illumina HiSeq 2000
Populus trichocarpa	SRR1030352	PE	11.8G	Illumina HiSeq 2000
Sorghum bicolor	ERR886716	PE	4.7G	Illumina HiScanSQ

Solanum lycopersicum	SRR2923343	PE	5.9G	Illumina HiSeq 2000
	SRR3095793	PE	1.9G	Illumina HiSeq 2000
Triticum aestivum	SRR1542404	PE	16.3G	Illumina HiSeq 2000
	SRR1542405	PE	15.2G	Illumina HiSeq 2000
	SRR2306548	PE	5.5G	Illumina HiSeq 2500
	SRR1228254	PE	3.7G	Illumina HiSeq 2000
Zea mays	SRR2069900	PE	5.4G	Illumina HiSeq 2500
	SRR762350	SE	6.9G	Illumina Genome Analyzer IIx

*PE: Paired end, **SE: Single end
