# Supplementary material for: PeTMbase: A Database of Plant Endogenous Target Mimics (eTMs)
Source: PLoS One. 2016 Dec 9;11(12):e0167698. doi: 10.1371/journal.pone.0167698 (PMC5148581; doi:10.1371/journal.pone.0167698)
Supplement: S2 Table — Reference genome assemblies utilized for short read mapping and alignment statistics associated with the transcriptome libraries. (PDF) [file pone.0167698.s002.pdf]

**S2 Table.** Reference genome assemblies utilized for short read mapping and alignment statistics associated with the transcriptome libraries.

| Reference Assembly | SRA ID | Number of input reads | Average input read length | Mapped reads % |
|---|---|---|---|---|
| *A. thaliana* - TAIR10 | ERR754084 | 17789212 | 202 | 79.16 |
| *B. distachyon* - v1.0 | DRR048605 | 29269012 | 202 | 91.11 |
| *G. max* - V1.0 | SRR3475914 | 17397088 | 202 | 94.07 |
| | SRR2079642 | 30643459 | 100 | 91.53 |
| | SRR1592302 | 26749369 | 100 | 95.16 |
| *H. vulgare* - ASM32608v1 | ERR674189 | 16791756 | 200 | 64.67 |
| *M. truncatula* - MedtrA17_4.0 | SRR1523070 | 47999588 | 200 | 86.38 |
| | SRR1523072 | 47434133 | 200 | 88.18 |
| | SRR1523077 | 45585568 | 200 | 88.72 |
| *Oryza sativa* - IRGSP-1.0 | SRR952242 | 15874767 | 202 | 95.98 |

| | | | | |
|---|---|---|---|---|
| | ERR687857 | 15781946 | 202 | 82.51 |
| *P. trichocarpa* - JGI2.0 | SRR1030352 | 65442430 | 180 | 96.45 |
| *S. bicolor* - Sorbi1 | ERR886716 | 23431944 | 202 | 94.83 |
| *S. lycopersicum* - SL2.50 | SRR2923343 | 29643874 | 200 | 84.47 |
| | SRR3095793 | 18881356 | 100 | 98.03 |
| *T. aestivum* - IWGSC1+popseq | SRR1542404 | 18110600 | 202 | 90.96 |
| | SRR1542405 | 81331219 | 200 | 91.34 |
| | SRR2306548 | 76134375 | 200 | 93.70 |
| | SRR1228254 | 27518448 | 200 | 86.36 |
| *Z. mays* - AGPv3 | SRR2069900 | 59966193 | 35 | 68.80 |
| | SRR762350 | 26544807 | 202 | 96.13 |
